# The identification of metabolites from gut microbiota in NAFLD via network pharmacology

**DOI:** 10.1038/s41598-023-27885-w

**Published:** 2023-01-13

**Authors:** Ki-Kwang Oh, Haripriya Gupta, Byeong Hyun Min, Raja Ganesan, Satya Priya Sharma, Sung Min Won, Jin Ju Jeong, Su Been Lee, Min Gi Cha, Goo Hyun Kwon, Min Kyo Jeong, Ji Ye Hyun, Jung A Eom, Hee Jin Park, Sang Jun Yoon, Mi Ran Choi, Dong Joon Kim, Ki Tae Suk

**Affiliations:** Center for Microbiome, Institute for Liver and Digestive Diseases, College of Medicine, Hallym University, Chuncheon, 24252 South Korea

**Keywords:** Microbiology, Gastroenterology

## Abstract

The metabolites of gut microbiota show favorable therapeutic effects on nonalcoholic fatty liver disease (NAFLD), but the active metabolites and mechanisms against NAFLD have not been documented. The aim of the study was to investigate the active metabolites and mechanisms of gut microbiota against NAFLD by network pharmacology. We obtained a total of 208 metabolites from the gutMgene database and retrieved 1256 targets from similarity ensemble approach (SEA) and 947 targets from the SwissTargetPrediction (STP) database. In the SEA and STP databases, we identified 668 overlapping targets and obtained 237 targets for NAFLD. Thirty-eight targets were identified out of those 237 and 223 targets retrieved from the gutMgene database, and were considered the final NAFLD targets of metabolites from the microbiome. The results of molecular docking tests suggest that, of the 38 targets, mitogen-activated protein kinase 8-compound K and glycogen synthase kinase-3 beta-myricetin complexes might inhibit the Wnt signaling pathway. The microbiota-signaling pathways-targets-metabolites network analysis reveals that *Firmicutes, Fusobacteria*, the Toll-like receptor signaling pathway, mitogen-activated protein kinase 1, and phenylacetylglutamine are notable components of NAFLD and therefore to understanding its processes and possible therapeutic approaches. The key components and potential mechanisms of metabolites from gut microbiota against NAFLD were explored utilizing network pharmacology analyses. This study provides scientific evidence to support the therapeutic efficacy of metabolites for NAFLD and suggests holistic insights on which to base further research.

The community of microorganisms inhabiting the human gut (gastrointestinal tract) is defined as the microbiota, which is estimated to be 100 trillion, including bacteria, viruses, fungi, and protozoa^1^. The gut microbiota is a significant element in human health and disease and variations in its diversity are associated with an unhealthy diet, medicines, and pathogenic infections as well as chronic kidney disease^2,3^. Notably, genetically engineered gut bacteria are significant therapeutic resources capable of producing beneficial metabolites for the treatment of chronic diseases such as cancer, autoimmune disorders, metabolic diseases, and beyond NAFLD^4^. An imbalance in gut microbiota can lead to the progression of some diseases, such as cancer, atherosclerosis, type 1 diabetes, and even nonalcoholic fatty liver disease (NAFLD)^5,6^. It has been suggested to have relatively stable and diverse distributions with a communal crucial microbiota, including the *Firmicutes* and *Bacteroidetes* phyla, as the key dominants^7^. The microbiota products are related to the occurrence and development of liver complications via diverse mechanisms, such as differential intestinal permeability, persistent inflammatory responses, and secretion of some short-chain fatty acids^8^. The microbiota products are related to the occurrence and development of liver complications via diverse mechanisms, such as differential intestinal permeability, persistent inflammatory responses, and secretion of some short-chain fatty acids^9^.

In particular, the gut-related microbiota converts exogenous and endogenous compounds into metabolites via the microbiota and nervous system^10^. These benefits of the cross-talk between microbiota and the gut can be exerted locally as well as in distant organs due to the systemic circulation of metabolites produced in the intestine^11^. Furthermore, the gut-liver axis is critical for understanding the mechanism of diverse liver diseases, such as NAFLD, nonalcoholic steatohepatitis (NASH), and the development and occurrence of cirrhosis^12^. For instance, the progression of NAFLD is related to lipopolysaccharide (LPS) produced by gram-negative bacteria inhabiting the gut^13^. Likewise, the gut microbiota converts choline into trimethylamine oxide, which exacerbates liver inflammation and damage^14,15^. This implies that the gut microbiota is critically related to liver diseases caused by inflammation. Over the past few years, the gut microbiota has been an increasingly significant therapeutic strategy for relieving NAFLD due to its great efficacy and low adverse effects^16^. The metabolites produced by gut microbiota are effective agents for the treatment of NAFLD^17^. Some microbiota-associated metabolites have been examined to determine either positive or negative effects on the development of NAFLD, even though the number of metabolites of gut microbiota is not completely clear^18^. Furthermore, the active metabolites of gut microbiota and their pharmacological mechanisms against NAFLD have not yet been reported. Hence, studies on active metabolites transformed by substrates and their mechanism of action should be better defined prior to clinical trials of proposed NAFLD treatments.

We suggest that the systematic methodology of network pharmacology can be used to unravel interactions of multiple components, for gut microbiota analysis, such as microbiota, signaling pathways, targets, and metabolites. Most recently, a report demonstrated that the gut microbiota have anti-fatigue effects by analyzing multiple targets via network pharmacology^19^. The development and occurrence of NAFLD are dependent on multiple factors that involve inherited characteristics as well as inconsistent microbiota distribution^20^. Therefore, network pharmacology would seem to be a very effective technology to explore the function of microbiota-related metabolites against diseases.

In this study, network pharmacology was utilized to investigate the analysis of a multi-factorial and very complex process, including key microbiota, signaling pathways, targets, and metabolites, in NAFLD. In parallel, we determined the key signaling pathways, targets, and metabolites to alleviate NAFLD. First, metabolites produced by the gut microbiome were identified utilizing a microbiome database, and metabolite-related targets were identified using cheminformatics. Then, NAFLD-related targets were retrieved via a bioinformatics database, and we identified the final targets among the metabolite-related targets and NAFLD targets. Second, we conducted a protein–protein interaction (PPI) network analysis, Kyoto encyclopedia of genes and genomes (KEGG) enrichment analysis, and gene ontology (GO) analysis. In key signaling pathways, we performed molecular docking test (MDT) to verify the most stable metabolites, which were identified by drug-likeness and toxicity in the in silico platform. Finally, we analyzed the microbiota-signaling pathways-targets-metabolites (MSTM) networks to identify the most significant components, microbiota, signaling pathways, targets, and metabolites from a holistic perspective. The workflow is represented in Fig. 1.

**Figure 1:**
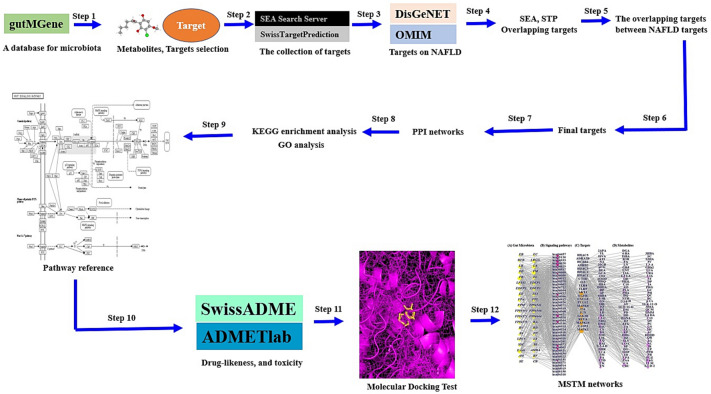
The workflow of this study.

## Methods

### Selection of gut microbiota metabolites and targets

The metabolites and targets of gut microbiota were retrieved by gutMGene v1.0 (http://bio-annotation.cn/gutmgene/) (Accessed on 2 April 2022). The Simplified Molecular Input Line Entry System (SMILES) formats of each metabolite were identified by PubChem (https://pubchem.ncbi.nlm.nih.gov/) (accessed on 3 April 2022).

### Identification of core targets against non-alcoholic fatty liver disease

The targets related to metabolites were identified through both similarity ensemble approach (SEA) (http://sea.bkslab.org/) (accessed on 4 April 2022)^21^ and SwissTargetPrediction (STP) (http://www.swisstargetprediction.ch/) (accessed on 4 April 2022)^22^ with the “*Homo sapiens*” setting. The overlapping targets between the SEA and STP databases were considered to be important targets for further analysis. In addition, NAFLD targets were obtained by DisGeNET (https://www.disgenet.org/) (accessed on 4 April 2022)^23^ and OMIM (accessed on 5 April 2022)^24^. Significant targets were identified among the metabolite-related targets and NAFLD targets. Then, the core targets were recognized between the significant targets and the gutMGene database.

### Construction of the protein–protein interaction network

The PPI network was constructed using R package and was based on final targets in STRING analysis (https://string-db.org/) (accessed on 6 April 2022). A target with the highest degree value in the PPI networks was considered a hub target to control the PPI network against NAFLD.

### Analysis of gene ontology and Kyoto encyclopedia of genes and genomes pathways of gut microbiota metabolites

GO analysis was performed to describe the functions of the targets, and consisted of molecular function (MF), biological function (BF), and cellular component (CC) analyses. The KEGG pathway enrichment analysis was used to understand the potential signaling pathways related to the final targets against NAFLD. The bubble plots are based on a rich factor defined as the gene ratio expressed differentially to the total target number in a signaling pathway^25^.

### The preparation of metabolites and targets for molecular docking testing

The metabolites associated with the key target were converted from the .sdf format from PubChem to.pdb format using PyMOL, and we obtained the .pdbqt format via AutoDock. The key target was identified in STRING through RCSB (https://www.rcsb.org/) (accessed on 6 April 2022). The.pdb format obtained by RCSB was converted into .pdbqt format by using AutoDock (http://autodock.scripps.edu/) (accessed on 6 April 2022).

### Molecular docking test of metabolites for the key target

The metabolites were docked with the key target utilizing AutoDock 4 by setting up 4 energy ranges and 8 exhaustiveness values as the defaults to acquire 10 different poses of the metabolites^26^. The center of the key target was x =  − 0.861, y = 2.109, z = 1.303. The active site grid box size was set to x = 40 Å, y = 40 Å, and z = 40 Å. Detailed information on 2D binding was generated by LigPlot + 2.2 (https://www.ebi.ac.uk/thornton-srv/software/LigPlus/download.html) (accessed on 7th April 2022)^27^. The threshold value of MDT was – 6.0 kcal/mol^28^ and a core metabolite with the lowest Gibbs free energy was selected on the metabolite-target complex in PyMOL.

### Evaluation of drug-likeness properties

The drug-likeness properties of the three metabolites were evaluated using SwissAMDE^29^ and the literature. Commonly, metabolites have hydrophilic properties and have low bioavailability; therefore, we identified their physicochemical properties through an in silico strategy.

### Toxicological evaluation by ADMETlab

One of key reason for failure of drug development is the lack of safety caused by some adverse effects: hERG blockers obstruct potassium channels^30^ and cause human hepatotoxicity^31^, Ames mutagenicity^32^, Skin sensitization^33^, Lethal Dose 50 (LD50) of acute toxicity^34^, and Drug Induced Liver Injury (DILI)^35^. Thus, we confirmed the six parameters by using ADMETlab platform^36^.

### Microbiota-signaling pathways-targets-metabolites network analysis

The MSTM networks were constructed as a size plot based on the degree value of each node. In the network plot, yellow circles (nodes) describe the gut microbiota; pink circles (nodes) display the signaling pathways; orange circles (nodes) represent the targets; and violet circles (nodes) represent the metabolites. The size of the yellow circles represents the total number of relationships with signaling pathways, metabolites, and targets; the size of pink circles represents the number of correlations with gut microbiota; the size of orange circles depicts the number of interactions with signaling pathways; and the size of violet circles describes the number of relationships with targets. The merged network was built using R Package.

## Results

### Acquisition of potential targets and metabolites of gut microbiota

We obtained 208 metabolites from the gutMgene microbiome database. The obtained targets and metabolites were considered significant components to analyze the therapeutic effects of the gut microbiota.

### Identification of 38 core targets from gut microbiota metabolites

**Figure 2:**
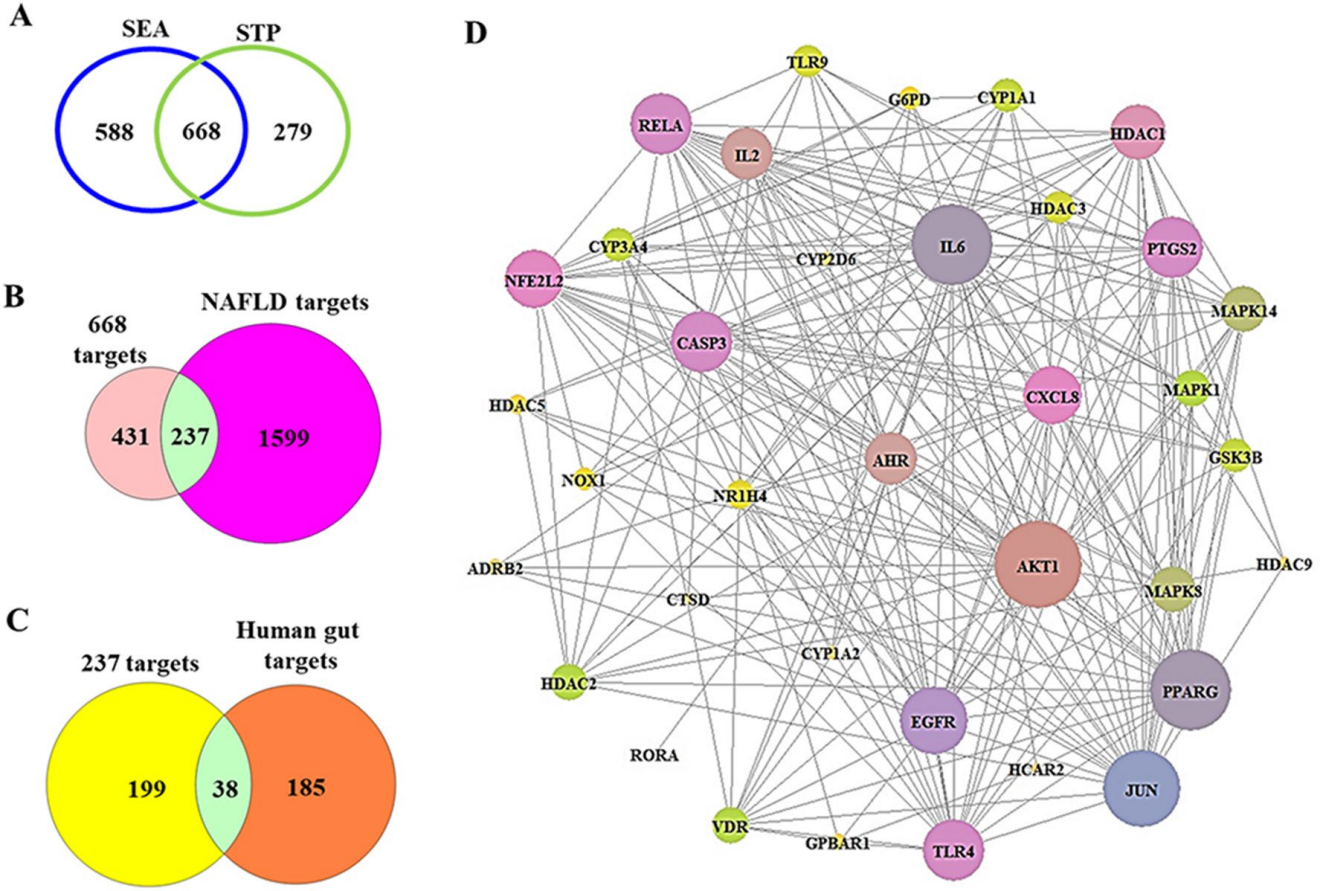
(**A**) The number of overlapping 668 targets between SEA and STP database. (**B**) The number of overlapping 237 targets between the 668 targets and NAFLD-related targets. (**C**) The number of the final overlapping 38 targets between the 237 targets and gut human targets. (**D**) The PPI networks (36 nodes and 237 targets).

A total of 208 metabolites were analyzed to search for their targets in silico in the SEA and STP databases. We identified 1256 targets from SEA and 947 targets from STP (Fig. 2A), and 668 targets were identified as overlapping targets between the two databases (Fig. 2B). A total of 237 targets among the 668 targets and 1836 NAFLD targets were identified; therefore, 38 core targets were obtained by analysis of the 223 targets (Fig. 2C).

### Protein–protein interaction network analysis

The PPI network consists of 36 nodes and 237 edges (Fig. 2D) in the 38 core targets, the size of which is based on the degree of value (Table 1). Two targets (ADRA2B and ST6GAL1) were not linked to one another in the 38 core targets. Based on the network map, a key target, AKT1, was defined as the uppermost target, followed by IL6, PPARG, JUN, and EGFR, further verifying the significant role of the target against NAFLD.

**Table 1:** The degree of value of PPI networks.

No.	Target	Degree of value	No.	Target	Degree of value
1	AKT1	28	19	VDR	12
2	IL6	26	20	CYP1A1	11
3	PPARG	26	21	CYP3A4	11
4	JUN	25	22	GSK3B	11
5	EGFR	22	23	HDAC3	10
6	CASP3	20	24	TLR9	10
7	PTGS2	20	25	NR1H4	9
8	RELA	20	26	G6PD	7
9	TLR4	20	27	NOX1	7
10	CXCL8	19	28	HDAC5	6
11	NFE2L2	19	29	ADRB2	5
12	HDAC1	18	30	GPBAR1	5
13	AHR	17	31	CYP1A2	4
14	IL2	17	32	CYP2D6	4
15	MAPK14	15	33	HDAC9	4
16	MAPK8	15	34	CTSD	3
17	HDAC2	12	35	HCAR2	3
18	MAPK1	12	36	RORA	1

HDAC5, histone deacetylase 5; ADRA2B, alpha-2B adrenergic receptor; HCAR2, hydroxycarboxylic acid receptor 2; ADRB2, adrenoceptor beta 2; HDAC3, histone deacetylase 3; HDAC2, histone deacetylase 2; HDAC1, histone deacetylase 1; CTSD, cathepsin D; IL2, interleukin 2; TLR4, toll-like receptor 4; TLR9, toll-like receptor 9; AKT1, AKT serine/threonine kinase 1; EGFR, epidermal growth factor receptor; CXCL8, C-X-C motif chemokine ligand 8; PTGS2, prostaglandin-endoperoxide synthase 2; MAPK8, mitogen-activated protein kinase 8; IL6, interleukin-6; JUN, jun proto-oncogene, AP-1 transcription factor subunit; GSK3B, glycogen synthase kinase-3 beta; RELA, RELA proto-oncogene, NF-KB subunit; MAPK14, mitogen-activated protein kinase 14; CASP3, caspase 3; MAPK1, mitogen-activated protein kinase 1.

### Identification of the 41 Kyoto encyclopedia of genes and genomes pathway enrichments and gene ontology enrichment analysis of the 3 components

**Figure 3:**
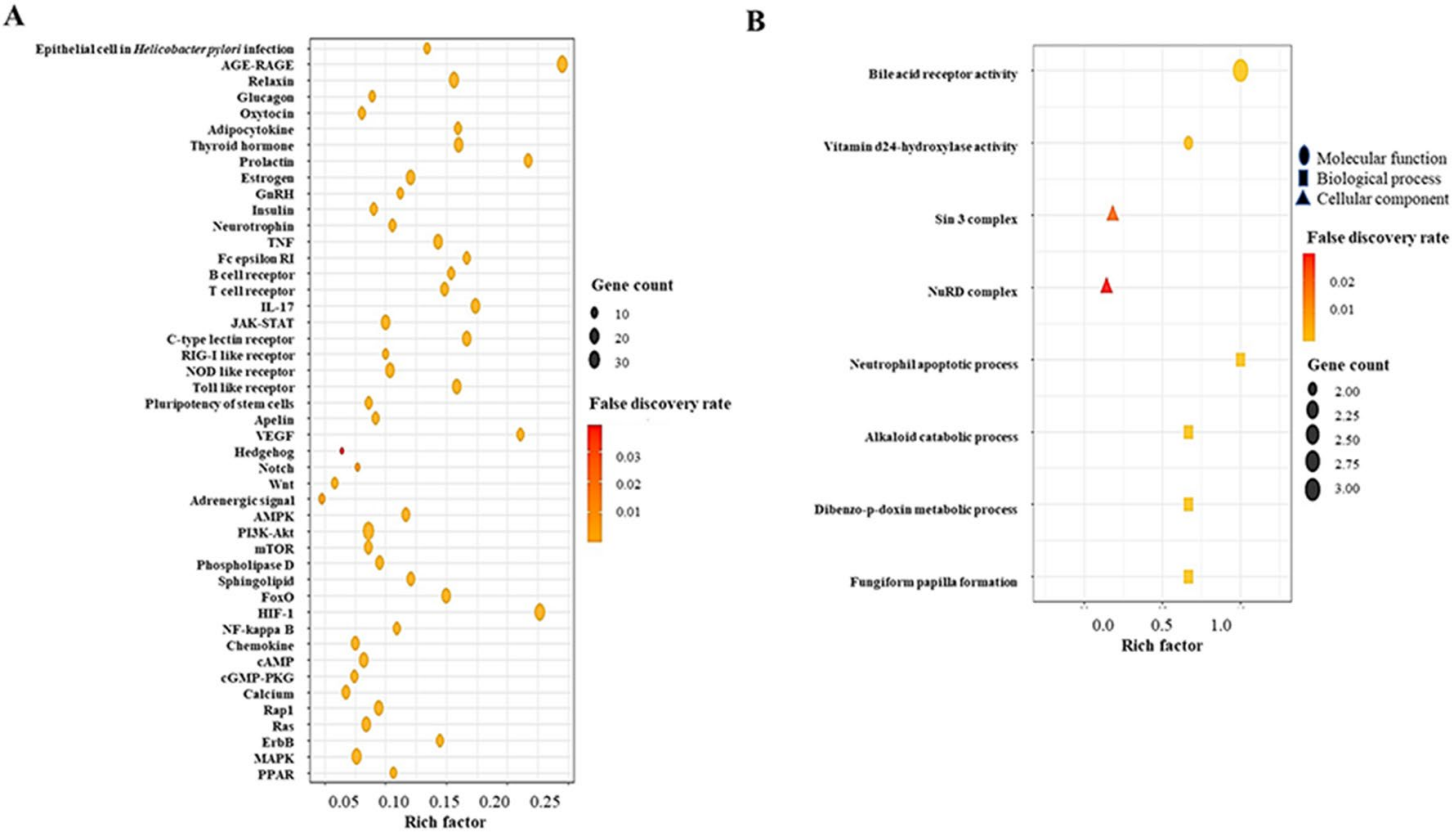
(**A**) KEGG enrichment analysis. (**B**) GO analysis.

To further evaluate the pharmacological mechanism of gut metabolites in the therapeutic strategy of NAFLD, the 38 core targets were investigated by KEGG pathway and GO enrichment analyses. The KEGG pathway enrichment analysis was based on signaling pathways (Table 2), the bubble size of which indicates the number of targets related to the pathway. The 41 signaling pathways of the KEGG pathway enrichment are represented in Fig. 3A, suggesting that the Wnt signaling pathway (Fig. 3B) might function as a potent inhibitive pathway of NAFLD. The GO enrichment analysis consisted of three components: molecular function (MF), biological process (BP), and cellular component (CC).

**Table 2:** The targets of 41 signaling pathways related to NAFLD.

KEGG ID and description	Target genes	False discovery rate
hsa04620: Toll-like receptor signaling pathway	MAPK1, AKT1, RELA, JUN, MAPK8, IL6, CXCL8, TLR4, TLR9, MAPK14	5.19E−10
hsa04657: IL-17 signaling pathway	MAPK1, RELA, GSK3B, JUN, MAPK8, IL6, PTGS2, CXCL8, MAPK14, CASP3	3.26E−13
hsa04933: AGE-RAGE signaling pathway in diabetic complications	MAPK1, AKT1, CASP3, RELA, JUN, MAPK8, IL6, CXCL8, MAPK14, NOX1	4.70E−13
hsa04668: TNF signaling pathway	MAPK1, AKT1, RELA, JUN, MAPK8, IL6, PTGS2, MAPK14, CASP3	3.02E−11
hsa04917: Prolactin signaling pathway	AKT1, RELA, GSK3B, MAPK8, MAPK14, MAPK1	6.52E−08
hsa04660: T cell receptor signaling pathway	MAPK1, AKT1, RELA, JUN, GSK3B, MAPK1, MAPK14, MAPK8	5.3E−10
hsa04625: C-type lectin receptor signaling pathway	MAPK1, AKT1, RELA, JUN, IL6, PTGS2, MAPK8, IL2, MAPK14	1.58E−11
hsa04012: ErbB signaling pathway	MAPK1, AKT1, JUN, GSK3B, EGFR, MAPK8	0.000000156
hsa04664: Fc epsilon RI signaling pathway	MAPK1, AKT1, MAPK14, MAPK8	0.0000462
hsa04066: HIF-1 signaling pathway	MAPK1, AKT1, RELA, EGFR, IL6, TLR4	0.000000552
hsa05120: Epithelial cell signaling in Helicobacter pylori infection	RELA, JUN, MAPK8, CXCL8, EFGR, MAPK14, CASP3	1.5E−09
hsa04722: Neurotrophin signaling pathway	MAPK1, AKT1, RELA, JUN, MAPK8, MAPK14	3.34E−08
hsa04662: B cell receptor signaling pathway	MAPK1, AKT1, RELA, JUN, GSK3B	0.00000319
hsa04370: VEGF signaling pathway	MAPK1, AKT1, PTGS2, MAPK14	0.0000278
hsa04071: Sphingolipid signaling pathway	MAPK1, AKT1, MAPK1, MAPK8, MAPK14, CTSD	0.000000861
hsa04068: FoxO signaling pathway	MAPK1, AKT1, IL6, MAPK8, EGFR, MAPK14	0.00000132
hsa04919: Thyroid hormone signaling pathway	MAPK1, AKT1, HDAC1, HDAC2, HDAC3, GSK3B	0.000000974
hsa04064: NF-kappa B signaling pathway	RELA, PTGS2, CXCL8, TLR4	0.0002
hsa04920: Adipocytokine signaling pathway	AKT1, RELA, MAPK8	0.0011
hsa04062: Chemokine signaling pathway	MAPK1, AKT1, RELA, GSK3B, CXCL8	0.00013
hsa04630: JAK-STAT signaling pathway	AKT1, IL2, IL6, EGFR	0.00092
hsa04926: Relaxin signaling pathway	MAPK1, AKT1, RELA, JUN, MAPK8, MAPK14, EGFR	6.55E−08
hsa04622: RIG-I-like receptor signaling pathway	RELA, MAPK8, MAPK14, CXCL8	0.0000547
hsa04621: NOD-like receptor signaling pathway	RELA, JUN, MAPK8, IL6, CXCL8, TLR4, MAPK14, MAPK1	2.15E−08
hsa04915: Estrogen signaling pathway	MAPK1, AKT1, JUN, EGFR, CTSD	0.0000312
hsa04024: cAMP signaling pathway	MAPK1, AKT1, RELA, JUN, MAPK8, HCAR2, ADRB2	0.00000112
hsa04910: Insulin signaling pathway	MAPK1, AKT1, GSK3B, MAPK8	0.000052
hsa04072: Phospholipase D signaling pathway	MAPK1, AKT1, EGFR, CXCL8	0.00071
hsa04150: mTOR signaling pathway	MAPK1, AKT1, GSK3B	0.0083
hsa04912: GnRH signaling pathway	MAPK1, JUN, MAPK8, EGFR, MAPK14	0.00000565
hsa04151: PI3K-Akt signaling pathway	MAPK1, AKT1, RELA, GSK3B, IL2, EGFR, IL6, TLR4	0.00000224
hsa04010: MAPK signaling pathway	MAPK1, AKT1, RELA, JUN, MAPK8, EGFR, MAPK14, CASP3	0.000000606
hsa04921: Oxytocin signaling pathway	MAPK1, JUN, EGFR, PTGS2	0.00073
hsa04371: Apelin signaling pathway	MAPK1, AKT1, HDAC5	0.0059
hsa04014: Ras signaling pathway	MAPK1, AKT1, RELA, MAPK8, EGFR	0.00031
hsa04022: cGMP-PKG signaling pathway	MAPK1, AKT1, ADRB2, ADRA2B	0.00094
hsa04261: Adrenergic signaling in cardiomyocytes	MAPK1, AKT1, MAPK14, ADRB2	0.00071
hsa04015: Rap1 signaling pathway	AKT1, MAPK14, EGFR	0.0019
hsa04550: Signaling pathways regulating pluripotency of stem cells	MAPK1, AKT1, MAPK14, GSK3B	0.00062
hsa04330: Notch signaling pathway	HDAC1, HDAC2	0.012
hsa04310: Wnt signaling pathway	JUN, MAPK8, GSK3B	0.0087

HDAC5, histone deacetylase 5; ADRA2B, alpha-2B adrenergic receptor; HCAR2, hydroxycarboxylic acid receptor 2; ADRB2, adrenoceptor beta 2; HDAC3, histone deacetylase 3; HDAC2, histone deacetylase 2; HDAC1, histone deacetylase 1; CTSD, cathepsin D; IL2, interleukin 2; TLR4, toll-like receptor 4; TLR9, toll-like receptor 9; AKT1, AKT serine/threonine kinase 1; EGFR, epidermal growth factor receptor; CXCL8, C-X-C motif chemokine ligand 8; PTGS2, prostaglandin-endoperoxide synthase 2; MAPK8, mitogen-activated protein kinase 8; IL6, interleukin-6; JUN, jun proto-oncogene, AP-1 transcription factor subunit; GSK3B, glycogen synthase kinase-3 beta; RELA, RELA proto-oncogene, NF-KB subunit; MAPK14, mitogen-activated protein kinase 14; CASP3, caspase 3; MAPK1, mitogen-activated protein kinase 1.

### Kyoto encyclopedia of genes and genomes pathway analysis

The Wnt signaling pathway out of the 41 KEGG pathways was the most significant mechanism and indicates the critical targets on the KEGG pathway enrichment diagram^37^.

### Molecular docking test

A total of 53 metabolites and three targets (JUN, MAPK8, and GSK3B) linked to the Wnt signaling pathway were identified via KEGG pathway enrichment analysis. MDT was performed to verify the binding affinity of each complex at the molecular level. AutoDockTools-1.5.6 software was used for MDT analysis; the docking scores are displayed in Supplementary Tables 1 and 2. The higher the negative docking score is, the more stable the complex is between the ligand and protein.

**Figure 4:**
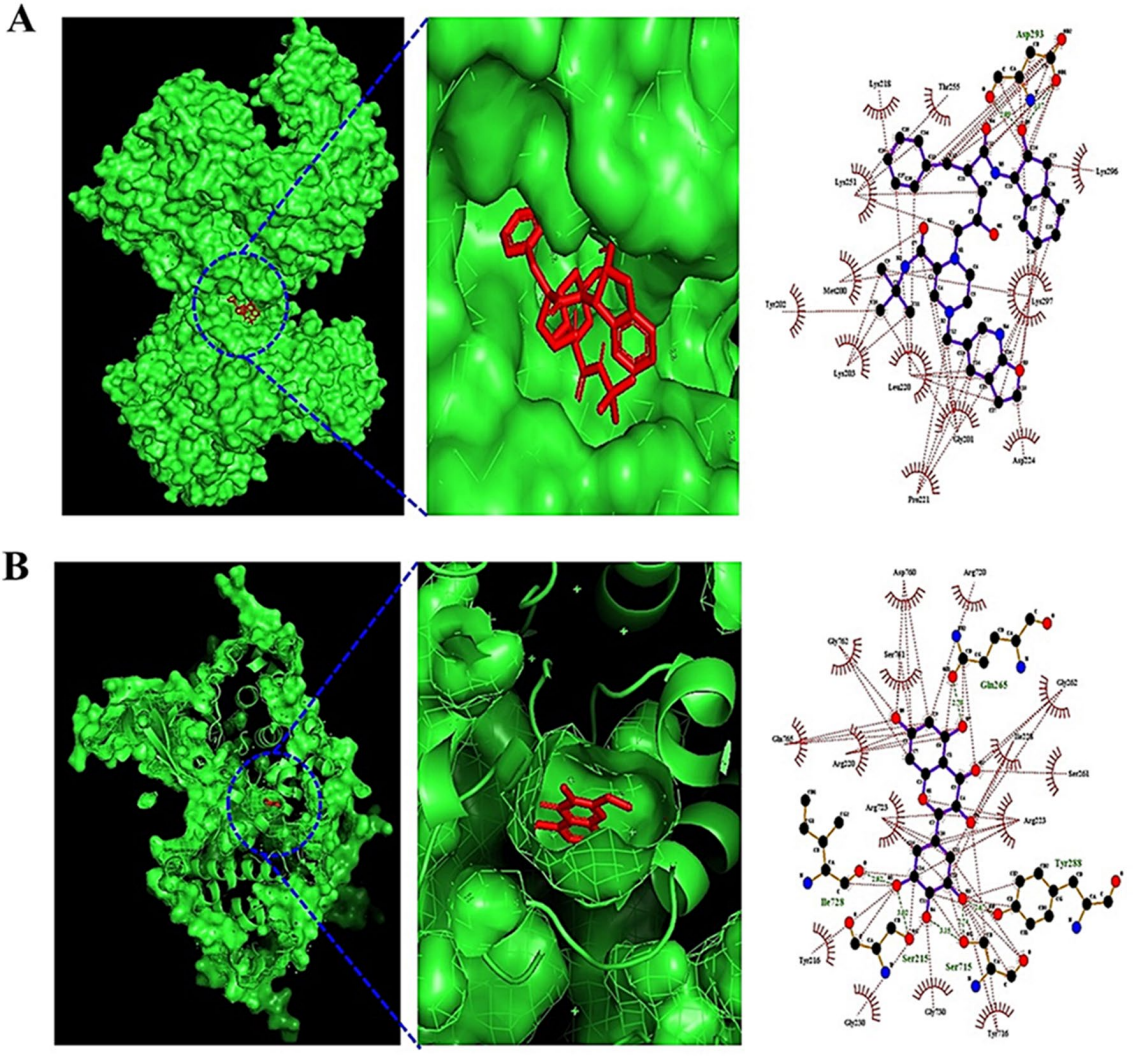
The molecular docking test on key targets of Wnt signaling pathway. (**A**) compound K-MAPK8 (PDB ID: 4YRB). (**B**) myricetin-GSK3B (PDB ID: 1J1B).

The cutoff of AutoDockTools-1.5.6 software is (< -6.0 kcal/mol), which can exert its efficacy on the target^28^. Between the 53 metabolites and 3 targets, the most stable complexes were JUN-platycodin D (− 9.0 kcal/mol), MAPK8-Compound K (− 8.5 kcal/mol), and GSK3B-myricetin (− 10.6 kcal/mol) (Fig. 4).

### Identification of drug-likeness properties in silico

The three metabolites (platycodin D, Compound K, and myricetin) were identified by the ADME parameters in silico. Platycodin D violated the drug-likeness properties characterized by Lipinski’s rule, including the topological polar surface area (TPSA) (cutoff value: < 140 Å^2^). The other two metabolites (Compound K and myricetin) had acceptable drug-likeness properties (Supplementary Table 3). Thus, we suggest that the two compounds can be metabolized by the gut microbiota and could be administered directly as new agents against NAFLD.

### Toxicological properties of the two metabolites

The possible toxicological properties of Compound K and myricetin were evaluated by the ADMElab online tool. Both were free of such attributes, which can be a hurdle for drug development (Supplementary Table 4).

### Identification of key components in the microbiota-signaling pathways-targets-metabolites network analysis

**Figure 5:**
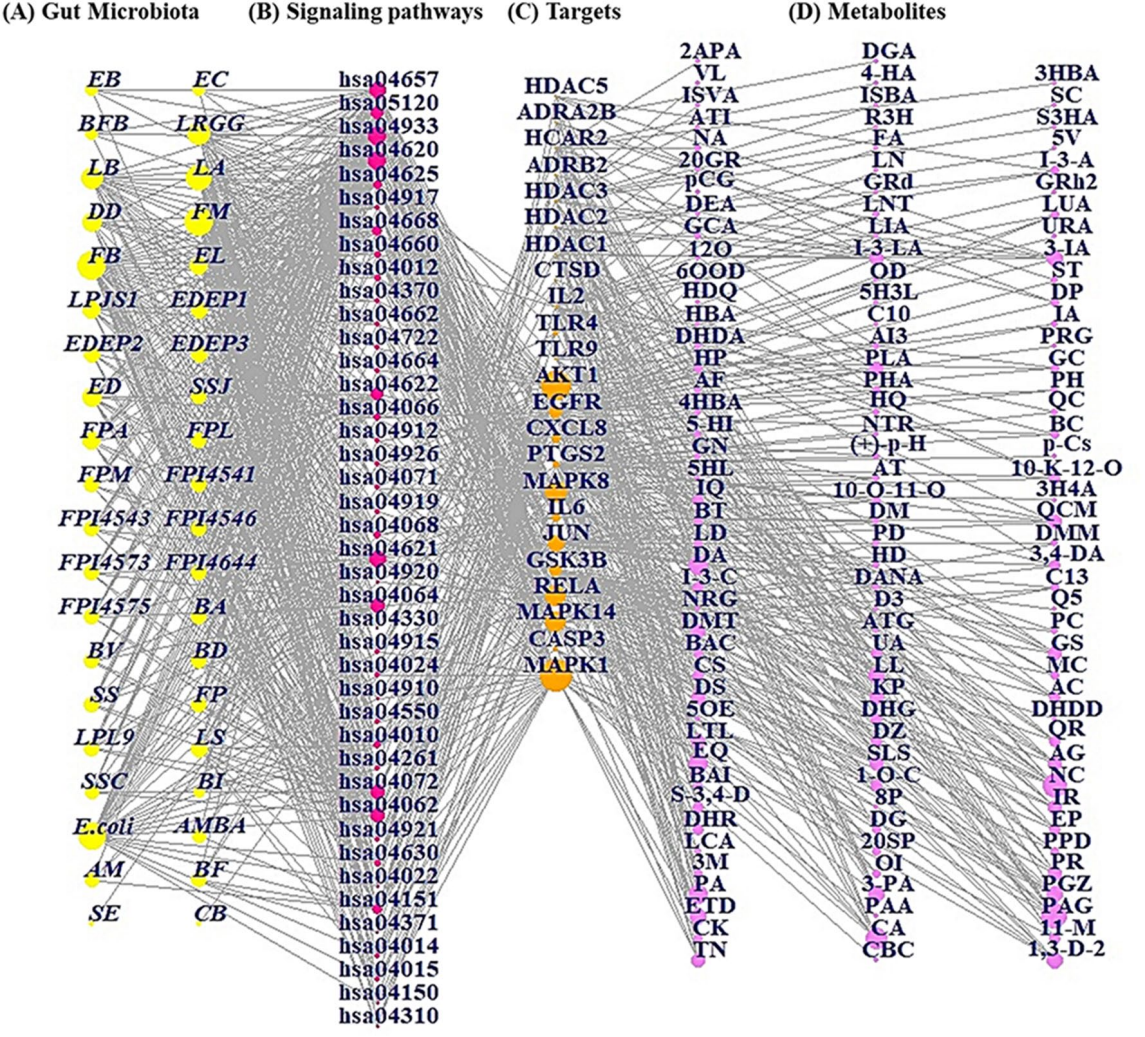
The MSTM networks (229 nodes and 1,044 edges). Yellow circle: gut microbiota; pink circle: signaling pathway; orange circle: target; violet circle: metabolite. (**A**) Microbiota. *Firmicutes: FM; Fusobacteria: FB; Escherichia coli; E.coli; Lactobacillus acidophilus ATCC 4357: LA; Lactobacillus rhamnosus GG: LRGG; Lactobacillus: LB; Dictyostelium discoideum: DD; Enterococcus durans M4-5: ED; Lactobacillus paracasei JS1: LPJS1; Faecalibacterium prausnitzii A2-165: FPA; Eubacterium limosum: EL; Enterococcus durans EP1: EDEP1; Enterococcus durans EP2: EDEP2; Enterococcus durans EP3: EDEP3; Lachnospiraceae: LS; Streptococcus salivarius JIM8772: SSJ; Faecalibacterium prausnitzii L2-6: FPL; Faecalibacterium prausnitzii M 21/2: FPM; Faecalibacterium prausnitzii CNCM I-4541: FPI4541; Faecalibacterium prausnitzii CNCM I-4543: FPI4543, Faecalibacterium prausnitzii CNCM I-4546: FPI4546; Faecalibacterium prausnitzii CNCM I-4573: FPI4573; Faecalibacterium prausnitzii CNCM I-4644: FPI4644; Faecalibacterium prausnitzii CNCM I-4575: FPI4575; Bifidobacterium adolescentis: BA; Bacteroides vulgatus: BV; Bacteroides distasonis: BD; Streptococcus salivarius: SS; Faecalibacterium prausnitzii: FP; Lactobacillus plantarum L9: LPL9; Bacteroides fragilis ATCC 23,745: BF; Streptococcus salivarius CIP102503: SSC; Akkermansia muciniphila ATCC BAA-835: AMBA; Faecalibacterium prausnitzii A2* < *U* + *2013* > *165: FPA2; Akkermansia muciniphila: AM; Eubacterium: EB; Enterococcus: EC; Bifidobacterium: BFB; Bacteroides: BI; Salmonella enterica: SE; Clostridium butyricum ATCC 19,398: CB.* (**B**) Signaling pathways. hsa04620: Toll-like receptor signaling pathway; hsa04657: IL-17 signaling pathway; hsa04933: AGE-RAGE signaling pathway in diabetic complications; hsa04668: TNF signaling pathway; hsa04917: Prolactin signaling pathway; hsa04660: T cell receptor signa;ing pathway; hsa05120: Epithelial cell signaling in *Helicobacter pylori* infection; hsa04722: Neurotrophin signaling pathway; hsa04662: B cell receptor signaling pathway; hsa04370: VEGF signaling pathway; hsa04071: Sphingolipid signaling pathway; hsa04068: FoxO signaling pathway; hsa04919: Thyroid hormone signaling pathway; hsa04064: NF-kappa B signaling pathway; hsa04920: Adipocytokine signaling pathway; hsa04062: Chemokine signaling pathway; hsa04630: JAK-STAT signaling pathway; hsa04926: Relaxin signaling pathway; hsa04622: RIG-I-like receptor signaling pathway; hsa04621: NOD-like receptor signaling pathway; hsa04915: Estrogen signaling pathway; hsa04024: cAMP signaling pathway; hsa04910: Insulin signaling pathway; hsa04072: Phospholipase D signaling pathway; hsa04150: mTOR signaling pathway; hsa04912: GnRH signaling pathway; hsa04151: PI3K-Akt signaling pathway; hsa04010: MAPK signaling pathway; hsa04921: Oxytocin signaling pathway; hsa04371: Apelin signaling pathway; hsa04014: Ras signaling pathway; hsa04022: cGMP-PKG signaling pathway; hsa04261: Adrenergic signaling in cardiomyocytes; hsa04015: Rap1 signaling pathway; hsa04310: Wnt signaling pathway; hsa04550: Signaling pathways regulating pluripotency of stem cells; hsa04330: Notch signaling pathway. (**C**) Targets. HDAC5: Histone deacetylase 5; ADRA2B: Alpha-2B adrenergic receptor; HCAR2: Hydroxycarboxylic acid receptor 2; ADRB2: Adrenoceptor Beta 2; HDAC3: Histone Deacetylase 3; HDAC2: Histone Deacetylase 2; HDAC1: Histone Deacetylase 1; CTSD: Cathepsin D; IL2: Interleukin 2; TLR4: Toll-like receptor 4; TLR9: Toll-like receptor 9; AKT1: AKT Serine/Threonine Kinase 1; EGFR: Epidermal Growth Factor Receptor; CXCL8: C-X-C Motif Chemokine Ligand 8; PTGS2: Prostaglandin-Endoperoxide Synthase 2; MAPK8: Mitogen-Activated Protein Kinase 8; IL6: Interleukin-6; JUN: Jun Proto-Oncogene, AP-1 Transcription Factor Subunit; GSK3B: Glycogen synthase kinase-3 beta; RELA: RELA Proto-Oncogene, NF-KB Subunit; MAPK14: Mitogen-Activated Protein Kinase 14; CASP3: Caspase 3; MAPK1: Mitogen-Activated Protein Kinase 1. (**D**) Metabolites. Phenylacetylglutamine: PAG; Naringenin chalcone: NC; Caffeic acid: CA; Phenylacetic acid: PA; Equol: EQ; Dihydroisoferulic acid: DA; 1,3-Diphenylpropan-2-ol: 1,3-D-2; Enterodiol: ETD; 3-Phenylpropionic acid: 3-PA; Pioglitazone: PGZ; Lunularin: LL; 3-Indolepropionic acid: 3-IA; Tretinoin: TN; Phloretin: PR; Icaritin: IR; Secoisolariciresinol: SLS; Apigenin: AG; Luteolin: LTL; Diosmetin: DS; Kaempferol: KP; Genistein: GS; Demethyltexasin: DMT; Quercimeritrin: QCM; Phenylalanine: PLA; Indole-3-lactic acid: I-3-LA; 11-Methoxycurvularin: 11-M; Dihydroresveratrol: DHR; Ethyl phenyllactate, (-)-: EP; Stilbene-3,4-diol: S-3,4-D; (S,R)-1-O-caffeoylglycerol: 1-O-C; Daidzein: DZ; Quercetin: QR; Acacetin: AC; Chrysin: CS; Urolithin A: UA; Indole-3-carboxylic acid: I-3-C; 3,4-Dihydroxyphenylacetic acid: 3,4-DA; Isoquercitrin: IQ; 10-Keto-12Z-octadecenoic acid: 10-K-12-O; Compound K: CK; 3-Methyloxindole: 3M; Oxindole: OI; (20S)-Protopanaxadiol: 20SP; Protopanaxadiol: PPD; Diosgenin: DG; Baohuoside I: BAI; Myricetin: MC; Baicalein: BAC; CHEBI:137478: C13; Levodopa: LD; Butyrate: BT; 10-Oxo-11-octadecenoic acid: 10-O-11-O; Baicalin: BC; Phloretic acid: PHA; HPLA: HP; Glycitein: GC; Dopamine: DP; Iuro-a: IA; Indole-3-acrylic acid: I-3-A; Dihydroglycitein: DHG; Leucocianidol: LCA; Ponciretin: PC; Danshensuan A: DANA; Hesperetin dihydrochalcone: HD; Platycodin D: PD; Didemethylmatairesinol: DMM; D-Mannose: DM; Acetic: AT; Genipin: GN; (+)-p-Hydroxyhydratropic acid: (+)-p–H; 5-HIAA: 5-HI; 4-Hydroxyphenylacetic acid: 4-HA; Hydroxyquercitrin: HQ; Quercitrin: QC; Acifran: AF; PhlP: PH; Dihydrocaffeic acid: DHDA; AI3-32,395: AI3; luro-a: IA; Serotonin: ST; Glycocholic acid: GCA; Lacto-N-tetraose: LNT; Nicotinic acid: NA; Colibactin: CBC; Palmitic acid: PAA; 8-Prenylnaringenin: 8P; 5-OH-Equol: 5OE; Dihydrogenistein: DHG; Dihydrodaidzein: DHDD; Arctigenin: ATG; Naringenin: NRG; DIF-3: D3; Q51617483: Q5; 3-Hydroxy-4-methoxybenzenepropanoic acid: 3H4A; 5-(Hydroxy-3-indolyl)lactic acid: 5HL; p-Cresol sulfate: p-Cs; Norathyriol: NTR; Phloroglucinol: PRG; Hydroumbellic acid: HDQ; CHEBI:10980: C10; Hydroquinone: HDQ; 5-hydroxyindole-3-lactic acid: 5H3L; 6′-OH-O-Dma: 6OOD; O-Desmethylangolensin: OD; 10-oxo-12Z-octadecenoic acid: 12O; Lithocholic acid: LIA; Ursodeoxycholic acid: URA; Deoxycholic acid: DEA; p-Cresol glucuronide: PCG; Ginsenoside-Rd: GRD; Ginsenoside Rh2: GRH; 20(R)-Ginsenoside Rh2: 20GR; LNnT: LN; Folic acid: FA; 5-(3,4-Dihydroxyphenyl)-valerolactone: 5V; Acetoin: ATI; (R)-3-Hydroxybutyrate: R3H; (S)-3-Hydroxybutyric acid: S3HA; Isovaleric acid: ISVA; Isobutyric acid: ISBA; Succinate: SC; Valerate: VL; 4-Hydroxybenzoic acid: 4HBA; 3-Hydroxybenzoic acid: 3HBA; 2-Acetoxypropanoic acid: 2APA; D-Glucuronic Acid: DGA.

The MSTM network analysis was performed using the R package with the STRING database, comprising 232 nodes (41 microbiota, 41 signaling pathways, 23 targets, and 127 metabolites) and 1047 edges of the network. The green circles represent the gut microbiota, the pink circles represent the signaling pathways, the orange circles depict the targets, and the violet circles describe the metabolites (Fig. 5). The connectivity between nodes indicates the direct relationships of the nodes. The greater the number of linked nodes is, the more significant the function of the microbiota, signaling pathways, targets, or metabolites. Then, we analyzed the degree of value using R package.

We discovered that *Firmicutes* and *Fusobacteria* are the most significant microbiota, with 586 degrees of value each, the Toll-like receptor signaling pathway is the most significant effector mechanism, with 33 degrees of value, MAPK1 is the uppermost target, with 34 degrees of value, and phenylacetylglutamine is the highest metabolite, with 10 degrees of value. The 4 components exhibited more relationships, suggesting that these components might be the most significant hallmarks in NAFLD.

## Discussion

We investigated the interaction between metabolites and gut microbiota via data-driven analysis. Previous research has suggested the use of gut microbiota in NAFLD treatment, but the details of the relevant metabolites and their targets remain unclear. Recently, network-based systems pharmacology has been used for diagnosis of various diseases and identification of target substances^38^. This study demonstrated that the relevant microbiome-derived metabolites might be detected by using network-based systems pharmacology, and the results of our study support the power of this approach.

In the PPI networks, AKT1, IL6, PPARG, JUN, and EGFR were defined as important targets. AKT inactivation attenuated NAFLD progression and liver tumorigenesis in mouse experiments^39^. The IL6 level was markedly increased in NAFLD patients, which can exacerbate its severity^40^. This implies that inactivation of IL6 might be a therapeutic strategy to alleviate NAFLD. Additionally, a study demonstrated that upregulation of PPARG can accelerate the progression of adipogenic hepatic steatosis^41^. In the NAFLD cellular sample, the expression of JUN was considerably elevated, suggesting that miR-139-5p overexpression is an indirect approach to dampen the JUN expression level^42^. An animal test suggested that epidermal growth factor receptor (EGFR) activation exacerbates the severity of NAFLD due to dysfunction of lipid metabolism^43^. Therefore, the five targets may be promising key targets for the treatment of NAFLD via gut microbiota metabolites.

The GO enrichment analysis results suggest that NAFLD targets of metabolites from gut microbiota are mainly related to bile acid receptor activity, vitamin D 24-hydroxylase activity, the Sin3 complex, nucleosome remodeling and the deacetylase (NuRD) complex, the neutrophil apoptotic process, alkaloid catabolic process, dibenzo-p-dioxin metabolic process, and fungiform papilla formation to relieve NAFLD. This analysis sheds light on the functions of metabolites in the treatment of NAFLD.

The results of the KEGG enrichment analysis indicate enrichment in inflammatory-related pathways, such as the IL-17 signaling pathway, AGE-RAGE signaling pathway, C-type lectin receptor signaling pathway, TNF signaling pathway, Toll-like receptor signaling pathway, T-cell receptor signaling pathway, epithelial cell signaling in Helicobacter pylori infection, the NOD-like receptor signaling pathway, neurotrophin signaling pathway, and prolactin signaling pathway. The targets of the key metabolites of gut microbiota associated with NAFLD are also related to inflammation. The relationships of the 10 significant pathways according to the FDR (false discovery rate < 0.05) are briefly discussed. IL-17 signaling pathway: IL-17 signaling aggravated the severity of NAFLD in mouse experiments due to the causal contribution of gut microbiota driving IL-17 production in damaged hepatocytes^44^. Advanced glycation end-products–receptor advanced glycation end-products (AGE-RAGE) signaling pathway: The upregulation of advanced glycation end-products (AGEs) accelerates the detrimental effects (liver injury, inflammation, and hepatic fibrosis) of NAFLD; therefore, a restrictive regime of AGEs might be a therapeutic strategy to relieve NAFLD^45^. C-type lectin receptor signaling pathway: C-type lectin is a hallmark to identify the stage of chronic liver disease, which is commonly upregulated in nonalcoholic steatohepatitis (NASH)^46^. It has been postulated that the overexpression of C-type lectin might induce excessive inflammation in hepatocytes. Tumor necrosis factor (TNF) signaling pathway: the expression level of TNF-α was increased in serum samples of NAFLD patients; in contrast, mice with deleted TNF receptors showed attenuated inflammation, steatosis, and fibrosis^47^. Toll-like receptor 7 (TLR7) dampened the development of NAFLD, and might be a potential treatment^48^. T-cell receptor signaling pathway: The dysregulation of T cells leads to the development of NAFLD, which results in cirrhosis and hepatocellular carcinoma^49^. Epithelial cell signaling in Helicobacter pylori infection: Helicobacter pylori infection might lead to NAFLD due to excessive inflammatory responses and insulin resistance^50^. NOD-like receptor signaling pathway: NLR induces the innate immune response to defend against foreign bodies, such as microbes or toxic chemicals, and the silencing of NLR can protect against cytokines^51^. Neurotrophin signaling pathway: The synthesis of brain-derived neurotrophic factor in the central nervous system indirectly enhances NAFLD via adiponectin^52^. Prolactin signaling pathway: Prolactin decreases lipid accumulation in hepatocytes, which ameliorates inflammation in the liver^53^. The rich factor (gene-ratio) results in our analysis showed the Wnt signaling pathway to have the lowest rich factor, indicating that the pathway might function as an inhibitive mechanism against NAFLD. Consistent with this result, Wnt antagonists have been shown to be a significant target for inhibiting the progression of NAFLD^54^.

Our study shows that Compound K and myricetin are promising antagonists that bind stably to MAPK8 and GSK3B in the Wnt signaling pathway, respectively. Compound K is a major metabolite of ginsenoside Rb1, which is converted by the gut microbiota^55^. Myricetin is a metabolite of myricitrin, which is transformed by *Escherichia* sp. 12, *Escherichia* sp. 33, and *Enterococcus* sp.45^56^. Furthermore, these metabolites have stable physicochemical properties in common in the systemic circulation and have low toxicity."^57^.

According to the MSTM networks, the results suggest that 41 microbiota constituents, 41 signaling pathways, 23 targets, and 125 metabolites might exert therapeutic efficacy against NAFLD. The *Firmicutes* phyla play significant roles in repressing the growth of pathogenic microbes, maintaining a constant immune system^58^. A group who consumed red wine combined with polyphenols had increased levels of *Fusobacteria* and *Firmicutes*, suggesting that the gut microbes might be significant players against cirrhosis^59^. Moreover, polyphenols play important roles in inhibiting hepatic fat accumulation, which has been confirmed by several in vitro experiments, in vivo tests, and clinical trials^60^. A finding which has been confirmed that both *Firmicutes* and *Fusobacteria* might exert desirable effects on NAFLD. MAPK inhibition attenuates obesity, insulin resistance, and steatosis in NAFLD^61^. With the highest degree of value of the metabolites of the gut microbiota, phenylacetylglutamine might be a biomarker to sign hepatic dysfunction^62^.

Our results in this study show that a holistic-based analysis, as integrated science, is a powerful tool for unraveling complex diseases and targets, as concluded by others^63^. Moreover, the associations and interactions between microbiota and complex chronic diseases can be better understood/elucidated utilizing network pharmacology concepts^64^.

## Conclusion

In summary, this study investigated the key metabolites of gut microbiota in treating NAFLD via a network pharmacology-based study. We revealed that Compound K and myricetin can function as antagonists of the Wnt signaling pathway by docking stably to MAPK8 (also known as JNK) and GSK3B. Our study provides crucial evidence that Compound K converted from ginsenoside Rb1 and myricetin converted from myricitrin can be administered orally as a therapeutic strategy against NAFLD. From a holistic viewpoint, *Firmicutes* and *Fusobacteria,* the Toll-like receptor signaling pathway, MAPK1, and phenylacetylglutamine might be important key components and distinctive features of NAFLD in MSTM networks. Thus, we suggest that a systemic approach to the analysis of metabolites of gut microbiota can be an effective methodology to screen therapeutic agents.

## Supplementary Information

Supplementary Tables.

## Data Availability

All data generated or analyzed during this study are included in this published article (and its Supplementary Information files).

## Abbreviations

BF: Biological function
CC: Cellular component
Gut: Gastrointestinal tract
KEGG: Kyoto encyclopedia of genes and genomes
LD50: Lethal dose 50
LPS: Lipopolysaccharide
LVEF: Left ventricular ejection fraction
MAFLD: Metabolic dysfunction-associated fatty liver disease
MDT: Molecular docking test
MF: Molecular function
MSTM: Microbiota-signaling pathways-targets-metabolites
NAFLD: Nonalcoholic fatty liver disease
NASH: Nonalcoholic steatohepatitis
NuRD: Nucleosome remodeling and deacetylase
PPI: Protein–protein interaction
T2DM: Type 2 diabetes mellitus
TMAO: Trimethylamine oxide
TPSA: Topological polar surface area

## References

[1] Valdes AM, Walter J, Segal E, Spector TD (2018). Role of the gut microbiota in nutrition and health. BMJ.

[2] Dogra SK, Doré J, Damak S (2020). Gut microbiota resilience: Definition, link to health and strategies for intervention. Front. Microbiol..

[3] Hobby GP, Karaduta O, Dusio GF, Singh M, Zybailov BL, Arthur JM (2019). Chronic kidney disease and the gut microbiome. Am. J. Physiol. Renal Physiol..

[4] Dosoky NS, May-Zhang LS, Davies SS (2020). Engineering the gut microbiota to treat chronic diseases. Appl. Microbiol. Biotechnol..

[5] Żółkiewicz J, Marzec A, Ruszczyński M, Feleszko W (2020). Postbiotics—A step beyond pre- and probiotics. Nutrients.

[6] Albhaisi SAM, Bajaj JS (2021). The influence of the microbiome on NAFLD and NASH. Clin. Liver Dis..

[7] Ahlawat S, Sharma KK (2021). Gut–organ axis: a microbial outreach and networking. Lett. Appl. Microbiol..

[8] Schwenger KJ, Clermont-Dejean N, Allard JP (2019). The role of the gut microbiome in chronic liver disease: The clinical evidence revised. JHEP Rep..

[9] Chen J, Vitetta L (2020). Gut microbiota metabolites in NAFLD pathogenesis and therapeutic implications. Int. J. Mol. Sci..

[10] Chen Y, Zhou J, Wang L (2021). Role and mechanism of gut microbiota in human disease. Front. Cell. Infect. Microbiol..

[11] Zheng D, Liwinski T, Elinav E (2020). Interaction between microbiota and immunity in health and disease. Cell Res..

[12] Wiest R, Albillos A, Trauner M, Bajaj JS, Jalan R (2017). Targeting the gut-liver axis in liver disease. J. Hepatol..

[13] Ohtani N, Kawada N (2019). Role of the gut–liver axis in liver inflammation, fibrosis, and cancer: A special focus on the gut microbiota relationship. Hepatol. Commun.

[14] Chu H, Duan Y, Yang L, Schnabl B (2019). Small metabolites, possible big changes: A microbiota-centered view of non-alcoholic fatty liver disease. Gut.

[15] He X, Ji G, Jia W, Li H (2016). Gut microbiota and nonalcoholic fatty liver disease: Insights on mechanism and application of metabolomics. Int. J. Mol. Sci..

[16] Jayakumar S, Loomba R (2019). Review article: emerging role of the gut microbiome in the progression of nonalcoholic fatty liver disease and potential therapeutic implications. Aliment. Pharmacol. Ther..

[17] Liu Q, Liu S, Chen L, Zhao Z, Du S, Dong Q (2019). Role and effective therapeutic target of gut microbiota in NAFLD/NASH. Exp. Ther. Med..

[18] Zhou J, Tripathi M, Sinha RA, Singh BK, Yen PM (2021). Gut microbiota and their metabolites in the progression of non-alcoholic fatty liver disease. Hepatoma Res..

[19] Muc-Wierzgon M, Martin-Pelaez S, Zhu H, Wang R, Hua H, Cheng Y (2022). Network pharmacology exploration reveals gut microbiota modulation as a common therapeutic mechanism for anti-fatigue effect treated with maca compounds prescription. Nutrients.

[20] Jennison E, Byrne CD (2020). The role of the gut microbiome and diet in the pathogenesis of non-alcoholic fatty liver disease. Clin. Mol. Hepatol..

[21] Keiser MJ, Roth BL, Armbruster BN, Ernsberger P, Irwin JJ, Shoichet BK (2007). Relating protein pharmacology by ligand chemistry. Nat. Biotechnol..

[22] Daina A, Michielin O, Zoete V (2019). SwissTargetPrediction: Updated data and new features for efficient prediction of protein targets of small molecules. Nucleic Acids Res..

[23] Piñero J, Ramírez-Anguita JM, Saüch-Pitarch J, Ronzano F, Centeno E, Sanz F (2020). The DisGeNET knowledge platform for disease genomics: 2019 update. Nucleic Acids Res..

[24] Hamosh A, Scott AF, Amberger JS, Bocchini CA, McKusick VA (2005). Online Mendelian Inheritance in Man (OMIM), a knowledgebase of human genes and genetic disorders. Nucleic Acids Res..

[25] Shi L, Yu L, Zou F, Hu H, Liu K, Lin Z (2017). Gene expression profiling and functional analysis reveals that p53 pathway-related gene expression is highly activated in cancer cells treated by cold atmospheric plasma-activated medium. PeerJ.

[26] Khanal P, Patil BM, Chand J, Naaz Y (2020). Anthraquinone derivatives as an immune booster and their therapeutic option against COVID-19. Nat. Prod. Bioprospect..

[27] Laskowski RA, Swindells MB (2011). LigPlot+: Multiple ligand-protein interaction diagrams for drug discovery. J. Chem. Inf. Model..

[28] Shityakov S, Förster C (2014). In silico predictive model to determine vector-mediated transport properties for the blood–brain barrier choline transporter. Adv. Appl. Bioinform. Chem..

[29] Daina A, Zoete V (2016). A BOILED-egg to predict gastrointestinal absorption and brain penetration of small molecules. ChemMedChem.

[30] Lamothe SM, Guo J, Li W, Yang T, Zhang S (2016). The human ether-a-go-go-related gene (hERG) potassium channel represents an unusual target for protease-mediated damage. J. Biol. Chem..

[31] Mulliner D, Schmidt F, Stolte M, Spirkl HP, Czich A, Amberg A (2016). Computational models for human and animal hepatotoxicity with a global application scope. Chem. Res. Toxicol..

[32] Xu C, Cheng F, Chen L, Du Z, Li W, Liu G (2012). In silico prediction of chemical ames mutagenicity. J. Chem. Inf. Model..

[33] Alves VM, Muratov E, Fourches D, Strickland J, Kleinstreuer N, Andrade CH (2015). Predicting chemically-induced skin reactions. Part I: QSAR models of skin sensitization and their application to identify potentially hazardous compounds. Toxicol. Appl. Pharmacol..

[34] Lei T, Li Y, Song Y, Li D, Sun H, Hou T (2016). ADMET evaluation in drug discovery: 15 Accurate prediction of rat oral acute toxicity using relevance vector machine and consensus modeling. J. Cheminform..

[35] Xu Y, Dai Z, Chen F, Gao S, Pei J, Lai L (2015). Deep learning for drug-induced liver injury. J. Chem. Inf. Model..

[36] Dong J, Wang NN, Yao ZJ, Zhang L, Cheng Y, Ouyang D (2018). Admetlab: A platform for systematic ADMET evaluation based on a comprehensively collected ADMET database. J. Cheminform..

[37] Kanehisa M, Furumichi M, Sato Y, Kawashima M, Ishiguro-Watanabe M (2022). KEGG for taxonomy-based analysis of pathways and genomes. Nucleic Acids Res..

[38] Gocho Y, Liu J, Hu J, Yang W, Dharia NV, Zhang J (2021). Network-based systems pharmacology reveals heterogeneity in LCK and BCL2 signaling and therapeutic sensitivity of T-cell acute lymphoblastic leukemia. Nat. Cancer.

[39] Jeong SH, Kim HB, Kim MC, Lee JM, Lee JH, Kim JH (2018). Hippo-mediated suppression of IRS2/AKT signaling prevents hepatic steatosis and liver cancer. J. Clin. Investig..

[40] Wieckowska A, Papouchado BG, Li ZZ, Lopez R, Zein NN, Feldstein AE (2008). Increased hepatic and circulating interleukin-6 levels in human nonalcoholic steatohepatitis. Am. J. Gastroenterol..

[41] Yu S, Matsusue K, Kashireddy P, Cao WQ, Yeldandi V, Yeldandi AV (2003). Adipocyte-specific gene expression and adipogenic steatosis in the mouse liver due to peroxisome proliferator-activated receptor γ1 (PPARγ1) overexpression. J. Biol. Chem..

[42] Jin SS, Lin CJ, Lin XF, Zheng JZ, Guan HQ (2022). Silencing lncRNA NEAT1 reduces nonalcoholic fatty liver fat deposition by regulating the miR-139–5p/c-Jun/SREBP-1c pathway. Ann. Hepatol..

[43] Choung S, Kim JM, Joung KH, Lee ES, Kim HJ, Ku BJ (2019). Epidermal growth factor receptor inhibition attenuates non-alcoholic fatty liver disease in diet-induced obese mice. PLoS ONE.

[44] Harley ITW, Stankiewicz TE, Giles DA, Softic S, Flick LM, Cappelletti M (2014). IL-17 signaling accelerates the progression of nonalcoholic fatty liver disease in mice. Hepatology.

[45] Wang J, Liu H, Xie G, Cai W, Xu J (2020). Identification of hub genes and key pathways of dietary advanced glycation end products-induced non-alcoholic fatty liver disease by bioinformatics analysis and animal experiments. Mol. Med. Rep..

[46] Schierwagen R, Uschner FE, Ortiz C, Torres S, Brol MJ, Tyc O (2020). The role of macrophage-inducible C-type lectin in different stages of chronic liver disease. Front. Immunol..

[47] Osawa Y, Kojika E, Hayashi Y, Kimura M, Nishikawa K, Yoshio S (2018). Tumor necrosis factor-α-mediated hepatocyte apoptosis stimulates fibrosis in the steatotic liver in mice. Hepatology Communications..

[48] Kim S, Park S, Kim B, Kwon J (2016). Toll-like receptor 7 affects the pathogenesis of non-alcoholic fatty liver disease. Sci. Rep..

[49] Hirsova P, Bamidele AO, Wang H, Povero D, Revelo XS (2021). Emerging roles of T cells in the pathogenesis of nonalcoholic steatohepatitis and hepatocellular carcinoma. Front. Endocrinol..

[50] Wijarnpreecha K, Thongprayoon C, Panjawatanan P, Manatsathit W, Jaruvongvanich V, Ungprasert P (2018). *Helicobacter pylori* and risk of nonalcoholic fatty liver disease: A systematic review and meta-analysis. J. Clin. Gastroenterol..

[51] Xu T, Du Y, Fang X-B, Chen H, Zhou D-D, Wang Y (2018). New insights into Nod-like receptors (NLRs) in liver diseases. Int. J. Physiol. Pathophysiol. Pharmacol..

[52] Wang YD (2021). New insight of obesity-associated NAFLD: Dysregulated “crosstalk” between multi-organ and the liver?. Genes Dis..

[53] Zhang P, Ge Z, Wang H, Feng W, Sun X, Chu X (2018). Prolactin improves hepatic steatosis via CD36 pathway. J. Hepatol..

[54] Wang S, Song K, Srivastava R, Dong C, Go GW, Li N (2015). Nonalcoholic fatty liver disease induced by noncanonical Wnt and its rescue by Wnt3a. FASEB J..

[55] Kim KA, Jung IH, Park SH, Ahn YT, Huh CS, Kim DH (2013). Comparative analysis of the gut microbiota in people with different levels of ginsenoside Rb1 degradation to compound K. PLoS ONE.

[56] Du LY, Zhao M, Xu J, Qian DW, Jiang S, Shang EX (2014). Identification of the metabolites of myricitrin produced by human intestinal bacteria in vitro using ultra-performance liquid chromatography/quadrupole time-of-flight mass spectrometry. Expert Opin. Drug Metab. Toxicol,.

[57] Wong AC, Levy M (2019). New approaches to microbiome-based therapies. mSystems..

[58] Jasirwan COM, Lesmana CRA, Hasan I, Sulaiman AS, Gani RA (2019). The role of gut microbiota in non-alcoholic fatty liver disease: Pathways of mechanisms. Biosci. Microbiota Food Health..

[59] Guohong-Liu QZ (2019). Characteristics of intestinal bacteria with fatty liver diseases and cirrhosis. Ann. Hepatol..

[60] Abenavoli L, Larussa T, Corea A, Procopio AC, Boccuto L, Dallio M (2021). Dietary polyphenols and non-alcoholic fatty liver disease. Nutrients.

[61] Cicuéndez B, Ruiz-Garrido I, Mora A, Sabio G (2021). Stress kinases in the development of liver steatosis and hepatocellular carcinoma. Mol. Metab..

[62] Delzenne NM, Knudsen C, Beaumont M, Rodriguez J, Neyrinck AM, Bindels LB (2019). Contribution of the gut microbiota to the regulation of host metabolism and energy balance: a focus on the gut-liver axis. Proc. Nutr. Soc..

[63] Barabási AL, Gulbahce N, Loscalzo J (2011). Network medicine: A network-based approach to human disease. Nat. Rev. Genet..

[64] Ding Y, Chen M, Wang Q, Gao L, Feng Y, Wang S (2020). Integrating pharmacology and microbial network analysis with experimental validation to reveal the mechanism of composite sophora colon-soluble capsule against ulcerative colitis. Evid.-Based Complement. Altern. Med..

