# Supplementary material for: The identification of metabolites from gut microbiota in NAFLD via network pharmacology
Source: Sci Rep. 2023 Jan 13;13:724. doi: 10.1038/s41598-023-27885-w (PMC9839744; doi:10.1038/s41598-023-27885-w)
Supplement: Supplementary file 1 — Supplementary Tables. [file 41598_2023_27885_MOESM1_ESM.docx]

**Supplementary Table 1.** The molecular docking test of metabolites on MAPK8 (PDB ID: 4YR8).

|  |  |  |  | **Grid box** | | **Hydrogen Bond Interactions** | **Hydrophobic Interactions** |
| --- | --- | --- | --- | --- | --- | --- | --- |
| **Protein** | **Ligand** | **PubChem ID** | **Binding energy(kcal/mol)** | **Center** | **Dimension** | **Amino acid Residue** | **Amino acid Residue** |
| MAPK8 (PDB ID: 4YR8) | Compound K | 5481990 | -8.5 | x=-50.617 | size_x = 40 | Asp293 | Lys296, Lys297, Asp224, |
|  |  |  |  | y=17.555 | size_y = 40 |  | Pro221, Gly201, Leu220, |
|  |  |  |  | z=61.646 | size_z = 40 |  | Lys203, Tyr202, Met200, |
|  |  |  |  |  |  |  | Lys251, Lys218, Thr255 |
|  |  |  |  |  |  |  |  |
|  | Protopanaxadiol | 9920281 | -8.3 | x=-50.617 | size_x = 40 | Gly199, Arg150 | Lys218, Trp222, Pro210, |
|  |  |  |  | y=17.555 | size_y = 40 |  | Phe215, Cys216, Arg174, |
|  |  |  |  | z=61.646 | size_z = 40 |  | Ala173, Leu172, Tyr202, |
|  |  |  |  |  |  |  | Lys225, Met200, Pro221, |
|  |  |  |  |  |  |  | Gln253, Glu217 |
|  |  |  |  |  |  |  |  |
|  | (20S)-Protopanaxadiol | 1.1E+07 | -7.1 | x=-50.617 | size_x = 40 | N/A | Glu217, Asn193, Arg208, |
|  |  |  |  | y=17.555 | size_y = 40 |  | Trp222, Cys216, Leu207, |
|  |  |  |  | z=61.646 | size_z = 40 |  | Asn212, Trp222, Pro210 |
|  |  |  |  |  |  |  | Lys218 |
|  |  |  |  |  |  |  |  |
|  | Acetic | 613145 | -6.9 | x=-50.617 | size_x = 40 | Gly201 | Pro254, Lys251, Ser307, |
|  |  |  |  | y=17.555 | size_y = 40 |  | Pro221, Met200, Leu220, |
|  |  |  |  | z=61.646 | size_z = 40 |  | Lys218, Gln290, Thr255, |
|  |  |  |  |  |  |  | Asp293 |
|  |  |  |  |  |  |  |  |
|  | 5-(Hydroxy-3-indolyl)lactic acid | 192215 | -6.3 | x=-50.617 | size_x = 40 | Gly199, Lys218, Glu217 | Pro221, Gln253, Phe215, |
|  |  |  |  | y=17.555 | size_y = 40 |  | Cys216, Pro254, Lys218, |
|  |  |  |  | z=61.646 | size_z = 40 |  | Trp222 |
|  |  |  |  |  |  |  |  |
|  | 1,3-Diphenylpropan-2-ol | 138478 | -6.3 | x=-50.617 | size_x = 40 | N/A | Phe215, Cys216, Gln253, |
|  |  |  |  | y=17.555 | size_y = 40 |  | Glu217, Lys218, Trp222, |
|  |  |  |  | z=61.646 | size_z = 40 |  | Pro221 |
|  |  |  |  |  |  |  |  |
|  | Genipin | 442424 | -5.7 | x=-50.617 | size_x = 40 | Cys245, Lys308 | Phe271, Leu241, Ile304, |
|  |  |  |  | y=17.555 | size_y = 40 |  | Pro244, Phe248, Asp305, |
|  |  |  |  | z=61.646 | size_z = 40 |  | Val303, Leu302 |
|  |  |  |  |  |  |  |  |
|  | Phenylacetylglutamine | 92258 | -5.7 | x=-50.617 | size_x = 40 | Trp222, Pro210, Asn193, | Val211, Pro210, Cys216, |
|  |  |  |  | y=17.555 | size_y = 40 | Glu217 | Arg208 |
|  |  |  |  | z=61.646 | size_z = 40 |  |  |
|  | 3-Hydroxy-4-methoxybenzenepropanoic acid | 2752054 | -5.7 | x=-50.617 | size_x = 40 | Lys225, Trp222, Glu217 | Cys216, Val211, Gln253, |
|  |  |  |  | y=17.555 | size_y = 40 |  | Pro254,Pro221 |
|  |  |  |  | z=61.646 | size_z = 40 |  |  |
|  | Ethyl phenyllactate, (-)- | 9877619 | -5.4 | x=-50.617 | size_x = 40 | Gly199, Gln253 | Phe215, Lys225, Cys216, |
|  |  |  |  | y=17.555 | size_y = 40 |  | Trp222, Lys218, Glu217 |
|  |  |  |  | z=61.646 | size_z = 40 |  |  |
|  | 10-Keto-12Z-octadecenoic acid | 24970825 | -5.1 | x=-50.617 | size_x = 40 | Gln253 | Lys250, Pro254, Lys251, |
|  |  |  |  | y=17.555 | size_y = 40 |  | Trp222, Lys218, Phe215, |
|  |  |  |  | z=61.646 | size_z = 40 |  | Cys216, Gly199, Met200, |
|  |  |  |  |  |  |  | Pro221 |

**Supplementary Table 2.** The molecular docking test of metabolites on GSK3B (PDB ID: 1J1B).

|  |  |  |  | **Grid box** | | **Hydrogen Bond Interactions** | **Hydrophobic Interactions** |
| --- | --- | --- | --- | --- | --- | --- | --- |
| **Protein** | **Ligand** | **PubChem ID** | **Binding energy(kcal/mol)** | **Center** | **Dimension** | **Amino acid Residue** | **Amino acid Residue** |
| GSK3B (PDB ID: 1J1B) | Myricetin | 5281672 | -10.6 | x=24.687 | size_x = 40 | Gln265, Tyr288, Ser715, | Arg720, Gly262, Ser261, |
|  |  |  |  | y=-0.129 | size_y = 40 | Ser215, Ile728 | Ile228, Arg223, Tyr716, |
|  |  |  |  | z=-21.218 | size_z = 40 |  | Gly730, Gly230, Tyr216, |
|  |  |  |  |  |  |  | Gln765, Arg723, Arg220, |
|  |  |  |  |  |  |  | Gly762, Ser761, Arg760 |
|  |  |  |  |  |  |  |  |
|  | Demethyltexasin | 5284649 | -10.0 | x=24.687 | size_x = 40 | Tyr788, Ser215, Ile728, | Gly230, Tyr216, Arg223, |
|  |  |  |  | y=-0.129 | size_y = 40 | Asp260, Gln265, Tyr288 | Arg720, Gly262,Arg220, |
|  |  |  |  | z=-21.218 | size_z = 40 |  | Ser261, Tyr716, Arg723, |
|  |  |  |  |  |  |  | Ser715, Ile228, Gly730 |
|  |  |  |  |  |  |  |  |
|  | Apigenin | 5280443 | -9.7 | x=24.687 | size_x = 40 | Ser715, Tyr288, Ile228, | Tyr788, Tyr716, Arg723, |
|  |  |  |  | y=-0.129 | size_y = 40 | Gln765, Ile728, Ser215 | Leu727, Arg720, Arg220, |
|  |  |  |  | z=-21.218 | size_z = 40 |  | Arg223, Ser761, Gly762, |
|  |  |  |  |  |  |  | Tyr216, Phe729, Gly730 |
|  |  |  |  |  |  |  |  |
|  | Daidzein | 5281708 | -9.4 | x=24.687 | size_x = 40 | Ser215, Tyr788, Asp260, | Gly230, Gln265, Arg723, |
|  |  |  |  | y=-0.129 | size_y = 40 | Tyr288 | Arg720, Ser261, Gly262, |
|  |  |  |  | z=-21.218 | size_z = 40 |  | Arg220, Arg223, Tyr716, |
|  |  |  |  |  |  |  | Ile228,Gly730, Ser715, |
|  |  |  |  |  |  |  | Ile728 |
|  |  |  |  |  |  |  |  |
|  | Chrysin | 5281607 | -9.3 | x=24.687 | size_x = 40 | Ile228, Ser715, Tyr288, | Gln765,Arg220, Arg223, |
|  |  |  |  | y=-0.129 | size_y = 40 | Ile728, Ser215 | Arg723, Tyr716, Tyr788, |
|  |  |  |  | z=-21.218 | size_z = 40 |  | Phe729, Leu727, Tyr216, |
|  |  |  |  |  |  |  | Gly762, Ser761, Arg760, |
|  |  |  |  |  |  |  | Arg720 |
|  |  |  |  |  |  |  |  |
|  | Genistein | 5280961 | -9.2 | x=24.687 | size_x = 40 | Ser215, Ile228, Ser715, | Gln765, Arg220, Arg223, |
|  |  |  |  | y=-0.129 | size_y = 40 | Tyr288, Ile728, Ser215 | Arg723, Tyr716, Tyr788, |
|  |  |  |  | z=-21.218 | size_z = 40 |  | Phe729, Leu727, Gly730, |
|  |  |  |  |  |  |  | Tyr216,Gly762, Ser761, |
|  |  |  |  |  |  |  | Asp760, Arg720 |
|  |  |  |  |  |  |  |  |
|  | Ponciretin | 25201019 | -8.9 | x=24.687 | size_x = 40 | Arg180, Arg96, Ala204 | Gly202, Ser203, Lys792, |
|  |  |  |  | y=-0.129 | size_y = 40 |  | Asp90, Leu88, Pro794, |
|  |  |  |  | z=-21.218 | size_z = 40 |  | Phe793, Phe67, Ile217, |
|  |  |  |  |  |  |  | Glu790, Val763 |
|  |  |  |  |  |  |  |  |
|  | Luteolin | 5280445 | -8.8 | x=24.687 | size_x = 40 | Leu588, Glu290, Ser703 | Phe567, Val267, Pro294, |
|  |  |  |  | y=-0.129 | size_y = 40 |  | Ser566, Val263, Phe293, |
|  |  |  |  | z=-21.218 | size_z = 40 |  | Gly702, Val587 |
|  |  |  |  |  |  |  |  |
|  | Equol | 91469 | -8.8 | x=24.687 | size_x = 40 | Ser215, Tyr788, Gln265, | Gly230, Gly730, Tyr288, |
|  |  |  |  | y=-0.129 | size_y = 40 | Asp260 | Arg223, Ile728, Arg720, |
|  |  |  |  | z=-21.218 | size_z = 40 |  | Arg220,Ser261, Arg723, |
|  |  |  |  |  |  |  | Tyr716, Gly262, Ile228, |
|  |  |  |  |  |  |  | Ser715, Gly230 |
|  |  |  |  |  |  |  |  |
|  | Naringenin | 932 | -8.8 | x=24.687 | size_x = 40 | Arg180, Ala204, Asp90, | Gly202, Lys792, Gln89, |
|  |  |  |  | y=-0.129 | size_y = 40 | Leu88 | Phe67, Pro794, Phe793, |
|  |  |  |  | z=-21.218 | size_z = 40 |  | Val763, Ser203, Glu790 |
|  |  |  |  |  |  |  |  |
|  | Quercetin | 5280343 | -8.7 | x=24.687 | size_x = 40 | Ala204, Arg180, Arg96, | Gly202, Ser203, Asn95, |
|  |  |  |  | y=-0.129 | size_y = 40 | Gln89, Asp90 | Val87, Phe67, Lys792, |
|  |  |  |  | z=-21.218 | size_z = 40 |  | Phe793, Pro794, Val763, |
|  |  |  |  |  |  |  | Glu790 |
|  |  |  |  |  |  |  |  |
|  | Urolithin A | 5488186 | -8.7 | x=24.687 | size_x = 40 | Tyr288, Ser715, Gln765, | Arg223, Tyr716, Ser261, |
|  |  |  |  | y=-0.129 | size_y = 40 | Ile228 | Gln265, Asp760, Gly262, |
|  |  |  |  | z=-21.218 | size_z = 40 |  | Ser761, Arg723, Gly762 |
|  |  |  |  |  |  |  |  |
|  | Acacetin | 5280442 | -8.6 | x=24.687 | size_x = 40 | Arg180, Ala204, Arg96 | Ser203, Gly202, Lys792, |
|  |  |  |  | y=-0.129 | size_y = 40 |  | Pro794, Leu88, Asp90, |
|  |  |  |  | z=-21.218 | size_z = 40 |  | Glu790, Phe67, Phe793, |
|  |  |  |  |  |  |  | Ile217, Val763 |
|  |  |  |  |  |  |  |  |
|  | Baicalein | 5281605 | -8.6 | x=24.687 | size_x = 40 | Gly702, Asn595 | Pro294, Ser566, Glu597, |
|  |  |  |  | y=-0.129 | size_y = 40 |  | Val587, Phe567, Leu588, |
|  |  |  |  | z=-21.218 | size_z = 40 |  | Val267 |
|  |  |  |  |  |  |  |  |
|  | Diosmetin | 5281612 | -8.5 | x=24.687 | size_x = 40 | Arg596, Glu597, Phe567 | Gly702, Asn595, Leu588, |
|  |  |  |  | y=-0.129 | size_y = 40 |  | Ser566, Ile296, Val267, |
|  |  |  |  | z=-21.218 | size_z = 40 |  | Pro294, Lys292 |
|  |  |  |  |  |  |  |  |
|  | Kaempferol | 5280863 | -8.5 | x=24.687 | size_x = 40 | Leu588, Glu290 | Ser566, Val267, Pro294, |
|  |  |  |  | y=-0.129 | size_y = 40 |  | Phe567, Phe293, Val263, |
|  |  |  |  | z=-21.218 | size_z = 40 |  | Gly702, Val587 |
|  |  |  |  |  |  |  |  |
|  | Dihydrogenistein | 9838356 | -8.1 | x=24.687 | size_x = 40 | Arg680, Glu597, Leu588, | Gly702, Pro294, Lys292, |
|  |  |  |  | y=-0.129 | size_y = 40 | Arg596, Ala704 | Phe567, Glu290, Ser703 |
|  |  |  |  | z=-21.218 | size_z = 40 |  |  |
|  | Dihydrodaidzein | 176907 | -8.0 | x=24.687 | size_x = 40 | Arg680, Ala704, Arg596 | Ser703, Lys705, Glu290, |
|  |  |  |  | y=-0.129 | size_y = 40 |  | Phe567, Pro294, Leu588, |
|  |  |  |  | z=-21.218 | size_z = 40 |  | Val587, Lys292, Gly702 |
|  |  |  |  |  |  |  |  |
|  | 5-OH-Equol | 9795113 | -7.9 | x=24.687 | size_x = 40 | Arg596, Glu597, Glu290 | Lys292, Pro294, Ile717, |
|  |  |  |  | y=-0.129 | size_y = 40 |  | Phe293, Val263, Gly702, |
|  |  |  |  | z=-21.218 | size_z = 40 |  | Phe567, Asn595 |
|  |  |  |  |  |  |  |  |
|  | 1,3-Diphenylpropan-2-ol | 138478 | -7.8 | x=24.687 | size_x = 40 | Leu588 | Val263, Phe293, Phe567, |
|  |  |  |  | y=-0.129 | size_y = 40 |  | Val267, Pro294, Ser566, |
|  |  |  |  | z=-21.218 | size_z = 40 |  | Ile296, Val587, Gly702 |
|  |  |  |  |  |  |  |  |
|  | Pioglitazone | 4829 | -7.7 | x=24.687 | size_x = 40 | N/A | Val267, Lys271, Ser566, |
|  |  |  |  | y=-0.129 | size_y = 40 |  | Pro294, Ile717, Val263, |
|  |  |  |  | z=-21.218 | size_z = 40 |  | Ser703, Glu290, Arg680, |
|  |  |  |  |  |  |  | Gly702, Lys292, Val587, |
|  |  |  |  |  |  |  | Leu588, Phe567 |
|  |  |  |  |  |  |  |  |
|  | Dihydroresveratrol | 185914 | -7.7 | x=24.687 | size_x = 40 | Ser203 | Phe267, Lys792, Pro794, |
|  |  |  |  | y=-0.129 | size_y = 40 |  | Gly202, Val763, Ile217, |
|  |  |  |  | z=-21.218 | size_z = 40 |  | Arg180, Glu790, Val87 |
|  |  |  |  |  |  |  |  |
|  | Lunularin | 181511 | -7.7 | x=24.687 | size_x = 40 | N/A | Val763, Glu790, Phe793, |
|  |  |  |  | y=-0.129 | size_y = 40 |  | Pro794, Val87, Phe67, |
|  |  |  |  | z=-21.218 | size_z = 40 |  | Gly202, Lys792, Ile217 |
|  |  |  |  |  |  |  |  |
|  | Phenylacetylglutamine | 92258 | -7.6 | x=24.687 | size_x = 40 | Gln765, Arg723, Gly262 | Ser761, Gln265, Asp760, |
|  |  |  |  | y=-0.129 | size_y = 40 |  | Arg223, Tyr216, Ile728, |
|  |  |  |  | z=-21.218 | size_z = 40 |  | Tyr288, Ser215, Ser715, |
|  |  |  |  |  |  |  | Ile228, Gly730, Ser261, |
|  |  |  |  |  |  |  | Arg220 |
|  |  |  |  |  |  |  |  |
|  | Enterodiol | 115089 | -7.4 | x=24.687 | size_x = 40 | Arg96, Gln89, Asp90, | Val763, Phe67, Gly202, |
|  |  |  |  | y=-0.129 | size_y = 40 | Leu88 | Asn95, Lys792, Pro794, |
|  |  |  |  | z=-21.218 | size_z = 40 |  | Glu97, Glu790, Phe793 |
|  |  |  |  |  |  |  |  |
|  | Secoisolariciresinol | 65373 | -7.1 | x=24.687 | size_x = 40 | Thr289, Thr732, Tyr788, | Pro712, Ala731, Gly730, |
|  |  |  |  | y=-0.129 | size_y = 40 | Gly230, Asn287 | Asn785, Asn787, Thr232, |
|  |  |  |  | z=-21.218 | size_z = 40 |  | Pro212, Tyr288 |
|  |  |  |  |  |  |  |  |
|  | DIF-3 | 3081033 | -7.0 | x=24.687 | size_x = 40 | Gln265, Arg723 | Gln765, Ser261, Arg223, |
|  |  |  |  | y=-0.129 | size_y = 40 |  | Tyr716, Ser715, Ile228, |
|  |  |  |  | z=-21.218 | size_z = 40 |  | Ser215, Leu727, Leu227, |
|  |  |  |  |  |  |  | Ile728, Tyr216, Gly762, |
|  |  |  |  |  |  |  | Ser761, Asp760, Arg220, |
|  |  |  |  |  |  |  | Arg720, Gly262 |
|  |  |  |  |  |  |  |  |
|  | Icaritin | 5318980 | -7.0 | x=24.687 | size_x = 40 | Arg220, Arg720, Tyr722, | Glu749, Pro755, Tyr640, |
|  |  |  |  | y=-0.129 | size_y = 40 | Ser647, Asp260 | Tyr721 |
|  |  |  |  | z=-21.218 | size_z = 40 |  |  |
|  |  |  |  |  |  |  |  |
|  | Arctigenin | 64981 | -6.6 | x=24.687 | size_x = 40 | N/A | Gln185, Tyr140, Tyr222, |
|  |  |  |  | y=-0.129 | size_y = 40 |  | Glu249, Ala143, Gly253, |
|  |  |  |  | z=-21.218 | size_z = 40 |  | Arg144, Ser147, Pro255 |
|  |  |  |  |  |  |  |  |
|  | Oxindole | 321710 | -6.3 | x=24.687 | size_x = 40 | Gly262 | Ser261, Asp260, Arg720, |
|  |  |  |  | y=-0.129 | size_y = 40 |  | Arg220, Asp760, Arg223, |
|  |  |  |  | z=-21.218 | size_z = 40 |  | Gly762, Arg723 |

**Supplementary Table 3.** The physicochemical properties of key metabolites from gut microbiota.

| **No.** | **Compounds** | **Lipinski Rules** | | | | **Lipinski's Violations** | **Bioavailability Score** | **TPSA(Å²)** |
| --- | --- | --- | --- | --- | --- | --- | --- | --- |
|  |  | **MW** | **HBA** | **HBD** | **MLog P** |  |  |  |
|  |  | **< 500** | **< 10** | **≤ 5** | **≤ 4.15** | **≤1** | **> 0.1** | **<140** |
| 1 | Compound K | 653.80 | 8 | 4 | 1.72 | 1 | 0.55 | 131.17 |
| 2 | Myricetin | 318.24 | 8 | 6 | -1.08 | 1 | 0.55 | 151.59 |

**Supplementary Table 4.** Toxicological properties of key metabolites from the gut microbiota.

| **Parameters** | | **Metabolite** | | |  |
| --- | --- | --- | --- | --- | --- |
|  |  |  |  |  |  |
|  | | **Compound K** | **Myricetin** | |  |
| **1) hERG blockers** | | Non-blockers | Non-blockers | |  |
| **2) Human Hepatotoxicity (H-HT)** | | Negative | Negative | |  |
| **3) Ames Mutagenicity** | | Negative | Negative | |  |
| **4) Skin sensitization, (r)LLNA** | | Negative | Negative | |  |
| **5) LD50 of acute toxicity** | | 3.403 mg/kg | 2.691 mg/kg | |  |
| **6) Drug Induced Liver Injury (DILI)** | | Negative | Negative | |  |
| 1) Category 0: Non-blockers; Category 1: Blockers; 2) Category 0: H-HT negative (-); Category 1: H-HT positive (+);3) Category 0: Ames negative (-); Category 1: Ames positive (+);4) Category 0: Non-sensitizer; Category 1: Sensitizer; 5) High-toxicity: 1~50 mg/kg; Toxicity: 51~500 mg/kg; low-toxicity: 501~5000 mg/kg; | | |  |  |  |
| 6) Category 0: DILI negative (-); Category 1: DILI positive (+). | | |  |  |  |
|  | | |  |  |  |
|  | | |  |  |  |
|  | | |  |  |  |
|  | | |  |  |  |
